# The offsetting relationship between hand grip strength and hypertension: A cross-sectional study from physically disabled over 50 years old in China

**DOI:** 10.1371/journal.pone.0300314

**Published:** 2024-06-05

**Authors:** Liya Xu, Xiaowan Cai, Yimin Zhang, Xu Wen, Tingting Sun

**Affiliations:** 1 College of Education, Zhejiang University, Hangzhou, China; 2 Key Laboratory of the Ministry of Education of Sports and Physical Health, Beijing Sport University, Beijing, China; 3 Faculty of Sports and Science, Beijing Sport University, Beijing, China; 4 China Institute of Sports and Health, Beijing Sport University, Beijing, China; International Institute of Health Management Research - New Delhi, INDIA

## Abstract

**Objectives:**

To explore the relationship between hand grip strength (HGS) and blood pressure in physically disabled individuals over 50 years old.

**Methods:**

The research adopts a cross-sectional survey, and the data comes from the “2022–2023 Physical Health Monitoring and Scientific and Technological Services for Physical Disabilities” jointly carried out by Beijing Sport University and China Disabled Sports Management Center. Select physically disabled individuals over 50 years old and collect physical fitness measurement data. HGS was measured and adjusted based on body weight and waist circumference, with standard normal conversion. The relationship between HGS and blood pressure was analyzed using multiple linear regression, and further logistic regression was used to analyze the relationship between standard HGS and the risk of abnormal blood pressure.

**Results:**

695 disabled individuals participated in the experiment, including 402 males (57.84%) and 293 females (42.16%). Multiple linear regression analysis found that for each standard deviation increase in the standardized *Z-value* of relative HGS, the systolic and diastolic blood pressure of male individuals decreased by 2.391 mmHg (*P* = 0.008) and 1.229 mmHg (*P* = 0.025); decreased by 2.336 mmHg (*P* = 0.026) and 1.585 mmHg (*P* = 0.008), respectively, for female. The increase in HGS reduced the risk of hypertension in physical disabilities in males [*OR* = 0.820 *95%CIs* (0.670, 0.952)] (*P* = 0.003) and females [*OR* = 0.735 *95%CIs* (0.472, 0.986)] (*P* = 0.007).

**Conclusion:**

The HGS of middle-aged and elderly physically disabled individuals negatively correlates with blood pressure, indicating the importance of increasing muscle strength (HGS) in preventing blood pressure.

## Introduction

Hand grip strength (HGS) refers to the muscle strength of the hand that contracts equally in length. Its strength can directly reflect the maximum resistance contraction ability of the forearm flexor muscle group and indirectly reflect the muscle strength level of the upper limbs, as well as the physical condition of the person [1]. HGS as a simple, economical, and scientific means of monitoring physical health is increasingly being valued by people. HGS is not only one of the important indicators reflecting a person’s physical strength and quality but also the basis for the normal functioning of the hand, which is closely related to the individual’s daily life. In addition, as HGS decreases with age, it can also predict disability rates, cancer rates, and even mortality rates [2–5]. The research on HGS and disease risk has gradually attracted attention. Previous studies have found that HGS can be used as a predictor of cardiovascular disease, cancer and all-cause death [5–7].

The physically disabled have a higher risk of HGS decline due to their generally older age, higher prevalence of chronic diseases, decreased social support, reduced social activities, decreased amount of exercise and other reasons. The decrease in HGS has a more prominent impact on this population’s daily living ability and quality of life. At present, HGS has become an important indicator for predicting the daily self-care ability of middle-aged and elderly physically disabled [8]. Meanwhile, HGS, as one of the five evaluation indicators for middle-aged and elderly frailty [9], is closely related to the nutritional status, bone density, life expectancy, and other health conditions of the middle-aged and elderly [10, 11].

Hypertension is the most common chronic disease. As a significant risk factor for cardio cerebral vascular disease, it has become a worldwide public health problem. The 2020 China Cardiovascular Disease Report shows that the incidence of hypertension in China has been increasing year by year, with approximately 245 million adults suffering from hypertension [12]. Hypertension has become a significant public health issue affecting residents’ health [13, 14]. Middle-aged and elderly physically disabled face high poverty rates and health-related challenges, making them a relatively marginalized group in society. The risk factors of chronic diseases such as hypertension may lead to cardiovascular diseases in the elderly with disabilities, which may coexist with disability and increase the health burden of the affected population. Studies have shown that compared to non-disabled, disabled individuals had a 2.30 times higher risk of hypertension [15]. In addition, based on the prevalence of hypertension changes in high-fat diets and limited exercise lifestyles, the prevalence of chronic diseases among people with disabilities is expected to continue to increase [16].

Physical activity ability has been confirmed as an important predictor of population death. HGS, as an important evaluation indicator of physical activity ability, has important predictive value for cardiovascular diseases, including hypertension, diabetes and cancer [17–19]. A cross-sectional study in China showed that the higher HGS among middle-aged and older women (aged ≥ 45 years), the lower their risk of developing hypertension [20]. However, data from the NHANES survey of 4597 adults showed a positive correlation between HGS and the risk of adult hypertension, with an OR value of 1.24 [21]. The relationship between HGS and blood pressure may still be controversial among different researches [22].

At present, more attention is paid to the relationship between HGS and blood pressure in the general population, and less attention is paid to the group with physical disabilities. Focusing on the relationship between blood pressure and HGS in the group with physical disabilities can provide a basis for the development of hypertension prevention and intervention strategies for individuals with physical disabilities and help them better understand and prevent diseases. To better verify the relationship between HGS and blood pressure in the population with physical disabilities, this study selected middle-aged and elderly physically disabled as the research subjects, analyzed the relationship between HGS and blood pressure, and provided a basis for formulating hypertension prevention and intervention strategies for the physically disabled. The research hypothesis of this study is that there is a negative correlation between HGS and blood pressure in middle-aged and elderly individuals with disabilities.

## Methods

### Data source

The experimental data came from the “2022–2023 Physical Health Monitoring and Scientific and Technological Services for Physical Disabilities” jointly by Beijing Sport University and China Sports Management Center for the Disabled. The study selected 695 middle-aged and elderly physically disabled individuals over 50 years old as the research subjects. The research ethics have been approved by Beijing Sport University.

Inclusion criteria: a) disability type belongs to physical disability; b) involved in dysfunction or deformity, without amputation or disability; c) able to sit, stand, and walk independently.

Exclusion conditions: a) there has been a major illness recently and cannot participate in this test; b) disability years ≤ 2 years; c) individuals who are unable to take care of themselves and engage in activities on their own; d) having amputations; e) post-stroke patients.

Collected routine indicators such as height, weight, and waist circumference of subjects according to unified requirements. All information and collection had been approved by the patient’s written informed consent.

### Data collection

Blood pressure was measured by the Key Laboratory of the Ministry of Education of Sports and Physical Health laboratory staff using the Omron upper arm sphygmomanometer according to the unified operating process. Subjects should avoid smoking, drinking caffeinated beverages, or exercising within 30 minutes before the measurement. Each participant should rest for 10 minutes before taking two measures, with an interval of 1 minute. The average of the two measurements should be calculated as the individual blood pressure value for analysis. The definition of hypertension is based on the 2018 Chinese Hypertension Guidelines: blood pressure ≥ 140/90 mmHg (1 mmHg = 0.133 kPa) or taking anti-hypertensive drugs [23].

According to the established research protocol, all participants were required to use a calibrated Xin Dong Hua Teng dynamometer for HGS measurement. When measuring, the dominant hand should be determined first and measured twice in a standing position. The subject was required to continuously adjust the angle so that their index finger was at a 90° angle with the dynamometer’s handle, and the body posture should not be changed during the measurement process. This study uses the measured maximum value as the individual’s absolute HGS value. HGS testing is relatively simple and suitable for future confirmatory research.

### Data and statistical analysis

All data were analyzed using SPSS 24.0 software, with a statistically significant difference of *P*<0.05. Due to significant differences in HGS among disabled individuals of different genders, all data analysis was stratified by gender. The quantitative data of Normal distribution are described by mean±standard deviation (*x±s*), and the qualitative data are described by frequency and rate χ^2^ tests or independent two sample *t-tests*. To avoid the impact of weight on HGS, HGS was corrected by body mass index (BMI) and waist circumference, and relative HGS = absolute HGS/BMI/waist. Furthermore, relative HGS was transformed into standard HGS values by gender and age standard normal transformation (*Z* transformation, mean = 0, standard deviation = 1). Analyze the correlation between standard HGS and systolic and diastolic blood pressure through Spearman correlation analysis. The dependency relationship between standard HGS and blood pressure was analyzed through multiple linear regression analysis. Further, the relationship between standard HGS and the risk of blood pressure abnormalities was analyzed through logistic regression analysis.

## Results

### Population characteristics

A total of 695 middle-aged and elderly physically disabled individuals participated in the experiment, including 402 males (57.84%) and 293 females (42.16%). Comparing the basic characteristics of middle-aged and elderly physically disabled of different genders, it was found that the age composition of male and female middle-aged and elderly physically disabled was similar, and there was no statistically significant difference between the two groups. However, males exceeded females in height, weight, BMI, waist, systolic blood pressure, diastolic blood pressure, and HGS, and the differences were statistically significant (Table 1).

**Table 1:** Baseline characteristics of participants.

	Male (n = 402)	Female (n = 293)	Statistical value (*t* or χ^2^)	*P*
Age (year)	59.17±6.18	59.23±6.12	-0.138	0.890
Height (cm)	166.79±9.17	155.92±7.25	16.806	0.000
Weight (kg)	68.16±12.51	58.67±10.98	10.393	0.000
BMI (kg/m^2^)	24.43±3.81	23.16±4.33	6.893	0.008
Waist (cm)	91.06±10.56	84.35±10.84	8.130	0.000
Systolic pressure (mmHg)	133.82±17.53	128.97±18.54	3.514	0.000
Diastolic pressure (mmHg)	83.78±10.75	80.71±10.65	3.740	0.000
hypertension [Number(%)]	108(26.87)	51(17.41)	8.595	0.003
HGS (kg)	36.94±10.80	22.74±6.56	19.977	0.000

Sample middle-aged and elderly physically disabled who participated in the “2022–2023 Physical Health Monitoring and Scientific and Technological Services for Physical Disabilities” (n = 695).

BMI, body mass index; HGS, hand grip strength.

### Multiple regression analysis

Absolute HGS was considered a dependent variable in the present study for further statistical analysis (Table 2).

**Table 2:** Multiple linear regression analysis of absolute HGS of middle-aged and elderly physical disabilities.

Gender	Variable	Coefficient	*SE*	*t-value*	*P*	*VIF*
Male	Constant	5.760	7.396	7.568		
	Age	-0.215	0.211	-3.565	<0.001	1.122
	Height	0.164	0.067	6.943	<0.001	3.045
	BMI	0.450	0.199	2.423	0.016	2.796
	Waist	0.054	0.073	3.118	0.035	2.553
Female	Constant	5.230	7.700	5.542		
	Age	-0.230	0.174	-3.095	<0.001	1.334
	Height	0.103	0.061	6.655	<0.001	2.457
	BMI	0.410	0.089	8.257	0.015	2.612
	Waist	0.025	0.065	4.894	0.022	2.120

Multiple regression coefficients and the variance inflation factor (VIF) of age, height, BMI and waist for males and females, right hand grip as a response variable.

SE, standard error; VIF, variance inflation factors; BMI, body mass index; HGS, hand grip strength.

The estimated model for men was: HGS = 5.760–0.215 age+0.164 height+0.450 BMI+0.054 waist.

For men, the variance inflation factors (*VIF*) showed no evidence of a multicollinearity problem among the predictor variables age, height, BMI and waist. The multiple regression analysis coefficients also demonstrated a negative association between HGS and age (*P*<0.001). In contrast, a positive association was found between HGS and body height (*P*<0.001), BMI (*P* = 0.016), and waist (*P* = 0.035).

The estimated model for women was: HGS = 5.230–0.230 age+0.103 height+0.410 BMI+0.025 waist.

For women, VIF also showed no evidence of a multicollinearity problem among age, height, BMI and waist. The coefficients of the multiple regression analysis confirmed a negative association between HGS and age (*P*<0.001) and a positive association between HGS and body height (*P*<0.001), BMI (*P* = 0.015, and waist (*P* = 0.022).

### Relationship between relative HGS and blood pressure

**Fig 1:**
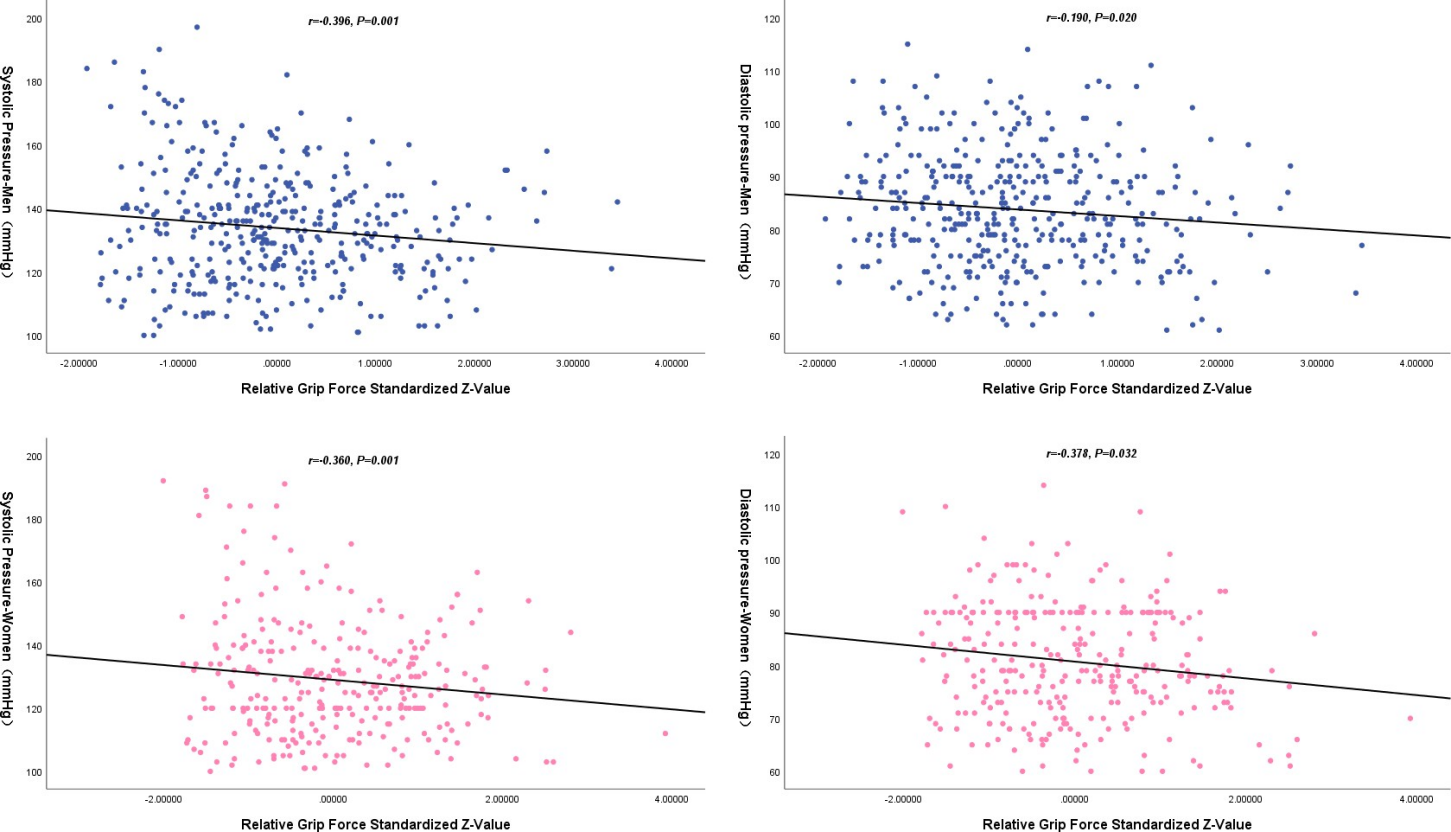
The relationship between the standardized Z-value of relative HGS and blood pressure.

Correct HGS through BMI and waist, and perform a standard normal transformation to generate relative HGS standardized *Z-values*. Analyze the relationship between standard HGS values and blood pressure (Fig 1).

Furthermore, through multiple linear regression analysis, it was found that for each standard deviation increase in the standardized *Z-value* of relative HGS, the systolic and diastolic blood pressure of male middle-aged and elderly physically disabled decreased by 2.391 mmHg (*P* = 0.008) and 1.229 mmHg (*P* = 0.025); for female, the average systolic and diastolic blood pressure decreases by 2.336 mmHg (*P* = 0.026) and 1.585 mmHg (*P* = 0.008), respectively (Table 3).

**Table 3:** Multiple linear regression analysis of the impact of relative HGS on blood pressure in middle-aged and elderly disabled individuals.

Gender	Variable	Systolic Pressure			Diastolic Pressure		
		Coefficient	SE	*P*-value	Coefficient	SE	*P*-value
Male	Age	0.578	0.832	0.521	1.425	0.499	0.752
	Relative HGS	-2.391	0.890	0.008	-1.229	0.547	0.025
Female	Age	0.451	0.962	0.632	0.751	0.652	0.320
	Relative HGS	-2.336	1.043	0.026	-1.585	0.597	0.008

SE, standard error; VIF, variance inflation factors; BMI, body mass index; HGS, hand grip strength.

Logistic regression analysis was conducted using hypertension as the dependent variable and adjusting for age. It was found that for each standard deviation increase in the standardized *Z-value* of relative HGS, the *OR* value for hypertension in male middle-aged and elderly physically disabled was 0.820 [*95%CIs* (0.670, 0.952)] (*P* = 0.003), while in female, the *OR* value was 0.735 [*95%CIs* (0.472, 0.986)] (*P* = 0.007) (Table 4). The increase in HGS is a protective factor for the occurrence of hypertension in middle-aged and elderly physically disabled individuals.

**Table 4:** Logistic regression analysis of the effect of relative HGS on blood pressure.

	OR(95%CI)	*P*
Gender		
Male	0.820(0.670,0.952)	0.003
Female	0.735(0.472,0.986)	0.007

## Discussion

In this study on the relationship between HGS and blood pressure in 695 middle-aged and elderly physically disabled aged 50 and above, it was found that HGS in males was more significant than in females, and there was a correlation between HGS and gender, age, height, BMI, and waist. The relative HGS of males and females was negatively correlated with systolic and diastolic blood pressure. Moreover, considering previous studies [24, 25], which presented a significant association between relative HGS and blood pressure, we included gender, age, height, BMI, and waist circumference in this association. We performed a separate analysis using a relative HGS. The results of our study presented a more significant association between relative HGS and hypertension compared with HGS and hypertension. Also, using relative HGS in the analysis improved the model fit of the analysis compared with when HGS was used.

The research results of this article were consistent with the conclusions of some scholars. A cross-sectional study of middle-aged and elderly people aged 45 and above in China showed that the greater HGS, the lower the risk of hypertension. HGS was independently associated with the risk of hypertension in women, but this association was not found in men [20]. Ji et al. found through cross-sectional data analysis of NHANES that the greater HGS, the higher diastolic blood pressure level, and the higher risk of hypertension, especially in obese men [21]. In addition, studies have shown that there is no correlation between HGS and hypertension risk among Korean women aged ≥ 20 years old [26]. Different research subjects, confounding factor adjustments, and choices of absolute and relative HGS values can lead to different results. Therefore, conducting long-term dynamic follow-up and analyzing the relationship between changes in HGS and blood pressure will help to better elucidate the relationship between HGS and blood pressure. Because HGS is a widely recommended measure of muscle strength [27], it is possible to consider that people with low HGS are less physically active, consequently increasing their risk of hypertension. Therefore, HGS could be used as a useful predictive indicator of hypertension in a clinical setting, as well as a reason to prescribe more exercise to those who exhibit low HGS. Another mechanism that could associate HGS with hypertension is the arterial structure. A previous study reported an improved structure in the brachial artery due to isometric handgrip exercise [28]. Accordingly, lower HGS could be associated with poor status of the brachial artery, which, in turn, could lead to hypertension. Subsequently, a type of exercise training could be recommended for those with hypertension, as well as those at risk of obtaining hypertension.

In addition, previous studies have considered the impact of weight on HGS when analyzing HGS and disease and health. Researchers often make weight corrections on HGS when analyzing absolute HGS, that is, generate relative HGS [20–22]. However, the multivariate analysis results of this study showed that HGS is not only related to weight but also to the waist, indicating the need for further adjustment of the waist, which may be more helpful in analyzing the correlation between HGS itself and disease or health. Therefore, based on weight adjustment, HGS also further adjusted the waist in this study. It is expected that more studies in the future will confirm the necessity of adjusting both weight and waist at the same time.

The HGS can be enhanced or maintained through training, while isometric training has been proven to be effective in reducing blood pressure, and continuous 12 weeks of moderate-intensity training can reduce blood pressure and improve hemodynamics in older women [29]. Meta-analysis also showed that exercise can effectively reduce blood pressure [30]. Although these exercise methods are not entirely the same, the relationship between exercise and blood pressure can also reflect the relationship between HGS and blood pressure. Meanwhile, a cross-sectional study of 795 Japanese older adults aged 60–89 speculated that subjects with beneficial effects on preventing skeletal muscle loss (maintaining HGS) may have active endothelial repair ability [31]. It is suggested that maintaining HGS will reduce the likelihood of new hypertension in the population.

If the disability condition is stable and there is no progressive development, people with physical disabilities can also achieve better health through rehabilitation exercise and physical fitness. However, the behavioural characteristics of physical disabilities in daily life, such as limited mobility and reduced activity, have led to a long-term lack of physical activity and physical exercise among this population, decreasing their HGS and significantly impacting their health. In the process of ageing, the incidence rate of hypertension is greatly increased, so it is necessary to evaluate the HGS of the middle-aged and elderly with disabilities and delay the incidence rate of hypertension.

### Limitations

Although our research has many advantages, we must also consider its limitations. Firstly, this study did not consider anti-hypertensive medicines and cardiovascular risk factors as confounding factors because it only focused on the correlation between current blood pressure and HGS. Future research can include the above factors. Moreover, Although the research findings can be extended to the physically disabled, they cannot represent more specific types of physical disabilities, such as amputees (who may not be able to measure HGS). Therefore, research in this area should continue to consider a wider range of populations and explore other methods of measuring muscle strength. For exploring the correlation, a longitudinal study or cohort study may be more able to explain the correlation between the two factors. In summary, future research can continue to study the physically disabled and analyze the relationship between their HGS and blood pressure at a deeper level from the perspective of a longitudinal study.

## Conclusion

This study found a negative correlation between HGS and blood pressure through the analysis of the relationship between HGS and blood pressure in middle-aged and elderly physically disabled, indicating the importance of increased muscle strength (HGS) in preventing blood pressure. Hypertension is a major public health problem faced by the middle-aged and elderly population, and in recent years, it has also shown a trend of youthfulness. Therefore, it is recommended that middle-aged and older adults with disabilities actively strengthen exercise and increase muscle strength under the guidance of professional clinical doctors to prevent or delay the occurrence and development of hypertension and increase social participation.
